# Machine-learning based prediction of appendicitis for patients presenting with acute abdominal pain at the emergency department

**DOI:** 10.1186/s13017-024-00570-7

**Published:** 2024-12-23

**Authors:** Anoeska Schipper, Peter Belgers, Rory O’Connor, Kim Ellis Jie, Robin Dooijes, Joeran Sander Bosma, Steef Kurstjens, Ron Kusters, Bram van Ginneken, Matthieu Rutten

**Affiliations:** 1 Diagnostic Image Analysis Group, Department of Medical Imaging, Radboud University Medical Center, Nijmegen, the Netherlands; 2 Emergency Department, Jeroen Bosch Hospital, ’s Hertogenbosch, the Netherlands; 3 Department of Radiology, Jeroen Bosch Hospital, ’s Hertogenbosch, the Netherlands; 4 Laboratory of Clinical Chemistry and Hematology, Jeroen Bosch Hospital, ’s Hertogenbosch, the Netherlands; 5 Department of Health Technology and Services Research, Technical Medical Centre, University of Twente, Enschede, the Netherlands; 6 Laboratory of Clinical Chemistry and Laboratory Medicine, Dicoon BV, location Canisius Wilhelmina Hospital, Nijmegen, the Netherlands

**Keywords:** Acute abdominal pain, Appendicitis, Machine learning, Artificial intelligence, Diagnostic follow-up, Clinical decision support, Emergency department

## Abstract

**Background:**

Acute abdominal pain (AAP) constitutes 5–10% of all emergency department (ED) visits, with appendicitis being a prevalent AAP etiology often necessitating surgical intervention. The variability in AAP symptoms and causes, combined with the challenge of identifying appendicitis, complicate timely intervention. To estimate the risk of appendicitis, scoring systems such as the Alvarado score have been developed. However, diagnostic errors and delays remain common. Although various machine learning (ML) models have been proposed to enhance appendicitis detection, none have been seamlessly integrated into the ED workflows for AAP or are specifically designed to diagnose appendicitis as early as possible within the clinical decision-making process. To mimic daily clinical practice, this proof-of-concept study aims to develop ML models that support decision-making using comprehensive clinical data up to key decision points in the ED workflow to detect appendicitis in patients presenting with AAP.

**Methods:**

Data from the Dutch triage system at the ED, vital signs, complete medical history and physical examination findings and routine laboratory test results were retrospectively extracted from 350 AAP patients presenting to the ED of a Dutch teaching hospital from 2016 to 2023. Two eXtreme Gradient Boosting ML models were developed to differentiate cases with appendicitis from other AAP causes: one model used all data up to and including physical examination, and the other was extended with routine laboratory test results. The performance of both models was evaluated on a validation set (*n* = 68) and compared to the Alvarado scoring system as well as three ED physicians in a reader study.

**Results:**

The ML models achieved AUROCs of 0.919 without laboratory test results and 0.923 with the addition of laboratory test results. The Alvarado scoring system attained an AUROC of 0.824. ED physicians achieved AUROCs of 0.894, 0.826, and 0.791 without laboratory test results, increasing to AUROCs of 0.923, 0.892, and 0.859 with laboratory test results.

**Conclusions:**

Both ML models demonstrated comparable high accuracy in predicting appendicitis in patients with AAP, outperforming the Alvarado scoring system. The ML models matched or surpassed ED physician performance in detecting appendicitis, with the largest potential performance gain observed in absence of laboratory test results. Integration could assist ED physicians in early and accurate diagnosis of appendicitis.

**Graphical abstract:**

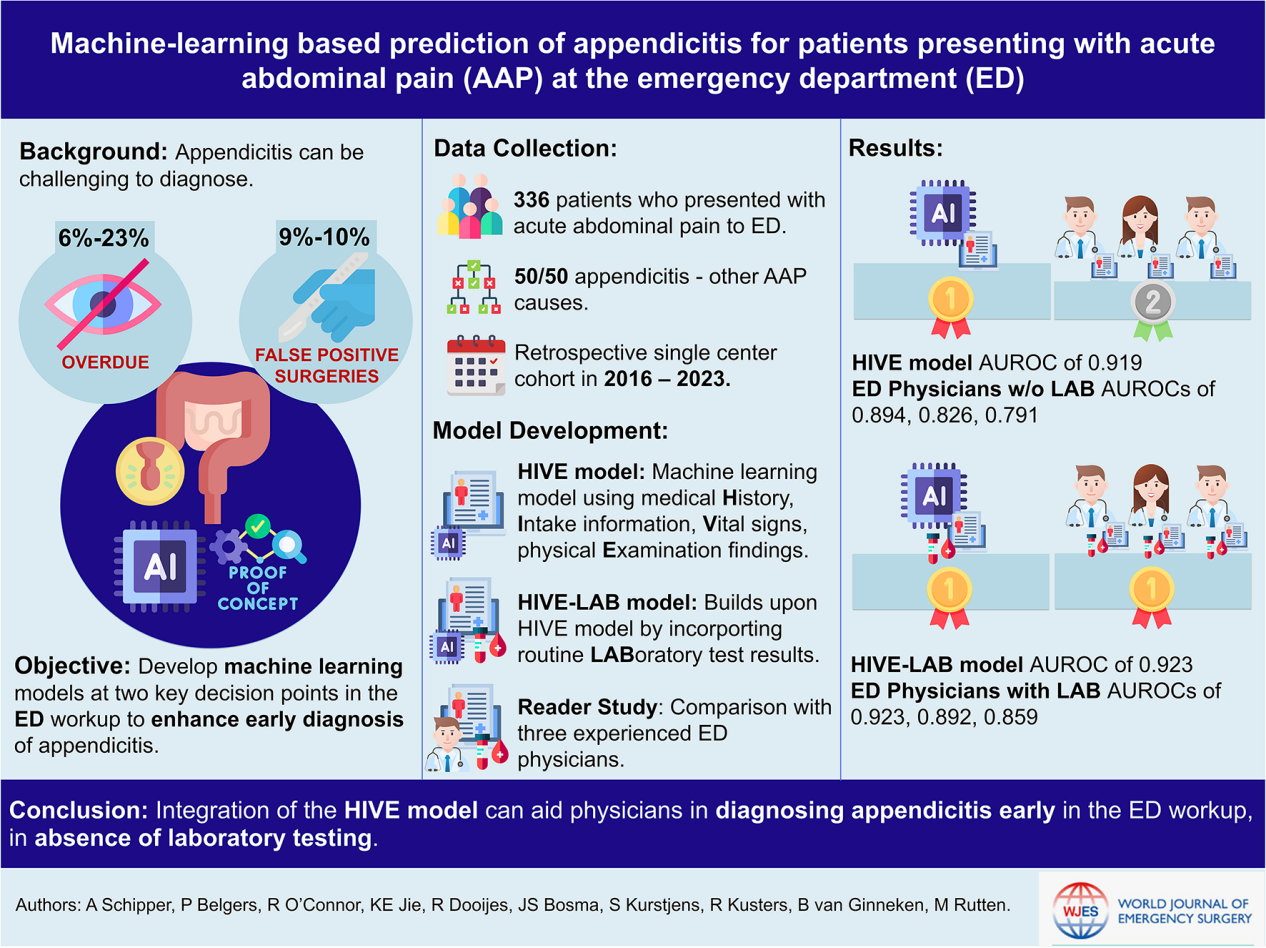

**Supplementary Information:**

The online version contains supplementary material available at 10.1186/s13017-024-00570-7.

## Introduction

Patients presenting with acute abdominal pain (AAP), comprising 5–10% of the emergency department (ED) population, experience prolonged lengths of stay, averaging over 4 h and exceeding 6 h for those undergoing computed tomography (CT) [1–3]. Causes range from benign and self-limiting to life-threatening conditions. Appendicitis, a prevalent cause of AAP and one of the most common emergency surgeries, usually manifests within a 24-hour time frame, although some cases are more chronic. Appendicitis can be subclassified into simple (non-perforated) and complex (gangrenous or perforated) conditions. Standard treatment involves an appendectomy, though conservative treatment, including pain control and antibiotics, is sometimes considered for simple appendicitis [4].

Patients with appendicitis usually present to the ED with AAP as their primary symptom. The diagnostic process for AAP in the ED in developed countries typically includes: (1) triage, (2) assessment of vital signs, (3) medical history and physical examination; (4) laboratory testing; (5) selective medical specialist consultations; and/or (6) imaging [1, 2]. In cases of suspected appendicitis, the aim of the diagnostic procedure is first to confirm or exclude appendicitis and second to stratify the condition [4].

Despite advancements in imaging techniques, which have significantly improved the accuracy of diagnosing appendicitis, distinguishing appendicitis from other AAP causes remains a significant clinical challenge [5]. In Northwestern Europe and the United States, the negative appendectomy rate - that is, the proportion of appendectomies performed on patients without appendicitis - ranges between 9% and 10.5% [6, 7]. The rate of missed appendicitis diagnoses ranges from 3.8 to 15.0% in children and from 5.9 to 23.5% in adults during ED visits [8]. These false-negative diagnoses are associated with higher rates of perforation, postoperative complications and interventions, as well as longer hospitalizations [6, 9]. Accurately recognizing the combination of signs and symptoms early in the ED workup is crucial to identify the risk of appendicitis, optimize the utility of diagnostic imaging, reduce the length of stay, and prevent negative surgical interventions.

To differentiate appendicitis from other AAP causes, various methods have been developed to stratify the risk of appendicitis and improve diagnostic accuracy and speed [e.g. 10–12]. The Alvarado score, introduced in 1986, is widely recognized as the best-known clinical scoring system for diagnosing appendicitis by combining signs, symptoms and laboratory test results [12, 13]. Although the literature has found the Alvarado score sensitive enough to rule out appendicitis, it lacks sufficient specificity, leading to a high false-positive rate [13, 14]. Machine learning (ML) models often show greater accuracy in predicting appendicitis compared to the Alvarado scoring system [15]. Unlike scoring systems for appendicitis that rely on a summation of points assigned to key clinical parameters, ML algorithms capture complex, nonlinear relationships and interactions among parameters. However, most ML models have primarily focused on reducing negative appendectomies by identifying candidates for surgery after the decision to operate has already been made [e.g. 8, 16–19]. Far fewer models have been applied to the broader AAP population [20–23]. Previous studies have not developed ML models designed to function as integrated decision-support systems within the ED workflow, specifically aimed at diagnosing appendicitis at the earliest possible stage. Furthermore, direct performance comparisons between ML models and ED physicians remain unexplored.

The aim of this study is to develop two novel ML models focusing on two critical decision points early in the ED workup: (1) evaluation based on intake information, vital signs, medical history and physical examination, and (2) subsequent assessment after laboratory testing. The performance of these models will be compared to that of ED physicians in a reader study and to the Alvarado score.

## Materials and methods

### Data collection

Pseudonymized data were retrospectively collected from 350 patients who presented with AAP and were registered with this complaint in the Dutch triage system at the ED of Jeroen Bosch Hospital, a Dutch teaching hospital in Den Bosch, between July 2016 and January 2023.

These patients’ visits to the ED are referred to as cases. No exclusions were made based on age, pregnancy status, comorbidities, medication use, or symptom presentation (see patient population details in Additional File 2). This inclusive approach aimed to reflect the diversity encountered in daily clinical practice and to develop ML models applicable to the entire AAP population. To limit the influence of any medications on the data, the first series of measurements from each ED visit was extracted. To train the model to differentiate appendicitis from other AAP causes, including those with similar clinical presentations, balanced subsampling was applied. This involved achieving equal numbers of appendicitis and other AAP cases. Additionally, among the other AAP cases, those suspected of appendicitis were balanced with those having non-specific or other AAP causes based on initial assessments by primary care physicians or triage nurses upon ED arrival. Balanced subsampling is a data preprocessing technique that enhances ML model performance on minority classes by balancing class distributions; it adjusts class frequencies without accounting for other parameters. No duplicate cases were introduced into our dataset during this process.

Other eligibility criteria included the availability of data from the initial patient evaluation relevant for building models at two key decision points in the ED workup. This included ED intake information, vital signs, medical history and physical examination findings from ED reports. In addition, blood and urine test results from standardized laboratory order sets, routinely requested for ED patients, were collected for these 350 cases. Detailed information on these parameters can be found in Supplemental Tables 1 A to 1E in Additional File 1. Cases were excluded if they had insufficient medical history or physical examination findings or were missing more than 70% of the laboratory tests results or vital signs (*n* = 14), a threshold chosen to balance the preservation of enough cases while minimizing missing parameters. This resulted in a final dataset of 336 eligible cases. The data extraction was performed using CTcue (IQVIA Nederland B.V., Amsterdam, the Netherlands), a privacy-by-design data extraction tool that automatically pseudonymizes patient data by redacting personally identifiable information and hashing patient IDs.

### Reference standard

The determination of ‘appendicitis’ versus ‘other AAP causes’ was based on three criteria: hospitalization, treatment received (e.g. surgery), and *International Classification of Diseases* 10th Revision (ICD-10) codes. This classification identified 167 cases, for which final pathology and/or radiology reports with confirmatory results were also available. Among the confirmed appendicitis cases, 109 patients underwent surgery, while 58 received conservative treatment. Each case was meticulously reviewed by a team of medical coders and, if necessary, the classification was adjusted after a patient’s hospitalization or surgery. Other AAP cases included 169 cases: 15 directly discharged from the ED and 154 patients lacking both ICD-10 codes for appendicitis and surgery. In these cases, appendicitis was neither suspected by ED physicians during their examinations nor confirmed by radiology or pathology reports. While appendicitis can sometimes resolve without treatment, such cases are rare, and there was no clinical evidence of appendicitis in these patients.

### Medical history and physical examination

Medical history and physical examination data provided in free-text entries in the ED reports were extracted from ED reports for each case. To structure this data, an initial annotation process was conducted by two researchers, who labeled all medical symptoms in 100 cases, resulting in 367 initial labels. The annotations were performed using annotation software Doccano (version 1.4) [24]. Labels with a prevalence of less than 5% were then reviewed by two ED physicians for their diagnostic value. Those deemed clinically unrelated to AAP causes were excluded, while others were grouped under overarching labels, reducing the total to 289. This final set of 289 labels was categorized into 73 parameters. These parameters included 50 binary parameters (e.g., presence of nausea) and 23 nominal parameters (e.g., location of pain). Another 236 cases were subsequently annotated using this structured framework.

### Model development

To estimate the probability of appendicitis at two key decision points in the ED workup of AAP, two ML models were constructed using the eXtreme Gradient Boosting (XGBoost) algorithm via the XGBoost package (version 2.0.3) [25]. The first model, coined the History Intake Vitals Examination (HIVE) model, used ED intake information, vital signs, medical history, and physical examination inputs. The second model, coined the HIVE-LAB model, was extended with laboratory test results. XGBoost was selected due to its strong performance in classification tasks and its native ability to handle missing data, a key consideration in both this study and daily clinical practice.

For model development, data from 336 cases were split into training and validation sets. 80% was used for training and hyperparameter tuning (*n* = 268: *n*_appendicitis_=133, *n*_other AAP causes_=135) and 20% for validation (*n* = 68: *n*_appendicitis_=34, *n*_other AAP causes_ =34). Repeated stratified 10-fold cross-validation was used for training and tuning to preserve the class distribution across folds, with the mean performance result across repetitions used for tuning the models. To handle binary and nominal parameters, a target-based encoding algorithm “CatBoost” (version 2.6.3) was used to encode binary and nominal parameters into numerical parameters representing statistical properties derived from the training data [26, 27]. Subsequently, both models were trained to optimize the area under the receiver operating characteristic curve (AUROC). Hyperparameters for the models were refined using Bayesian optimization through Optuna (version 3.6.1) [28], involving 100 trials to identify the optimal settings (see Hyperparameter settings XGBoost in Additional File 2). Model interpretation was performed using SHapley Additive exPlanations (SHAP) values by calculating the percentage contribution of each parameter to the prediction of the XGBoost models using the TreeSHAP algorithm (version 0.41.0) [29]. The TRIPOD checklist was followed to ensure increased transparency of the study’s methodology (Supplemental Table 2 in Additional File 1).

### Reader study - expert diagnosis

A reader study was conducted to compare the outcomes of the HIVE and HIVE-LAB models with the clinical performance of ED physicians using the same validation set (*n* = 68). Each case was presented in its original format, mimicking the electronic health record system, and independently evaluated by three ED physicians with one, five, and ten years of post-qualification experience. Each ED physician scored the likelihood of appendicitis for each case on a scale from 0 to 100, with 0 being ‘highly unlikely’ and 100 ‘very likely’. This scale mirrored the probability output of the models.

Initially, the physicians scored each case based on intake information, vital signs, medical history, and physical examination findings. Subsequently, they adjusted the likelihood score, if necessary, after evaluating the laboratory test results for the same case. This two-step evaluation process ensured that the assessments were comprehensive, reflective of real-world diagnostic practices, allowing for an assessment of the added value of the laboratory test results (See Example Case in Additional File 2).

### Alvarado scoring system

The Alvarado scoring system, also known as MANTRELS (Migration, Anorexia, Nausea-vomiting, Tenderness in right lower quadrant, Rebound pain, Elevation of temperature, Leukocytosis, Shift to the left), is a 10-point clinical scoring system developed for risk stratification of acute appendicitis for patients presenting with AAP (see Supplemental Table 3 in Additional File 1) [12]. A score of ≤ 4 is considered low risk, while a score of ≥ 7 high risk of appendicitis necessitating specialist consultation and/or further imaging [15]. This scoring system was applied to the validation set (*n* = 68) to compare performance with the HIVE model, the HIVE-LAB model, and ED physicians.

### Statistical analysis

Input parameters are presented as medians with interquartile ranges (IQR) or means with standard deviations (SD), depending on their distribution (Table 1, Supplemental Tables 1 A–1 C in Additional File 1). Differences in medians and means between cases with appendicitis and other AAP causes were assessed using Kruskal-Wallis tests or one-way ANOVA for continuous variables, and chi-square or Fisher’s Exact tests for categorical variables, as appropriate (Table 1, Supplemental Tables 1 A–1E in Additional File 1) [30]. DeLong’s test was employed to compare the AUROC values of the ML models, ED physicians, and the Alvarado score. Statistical significance was set at *p* < 0.05 (Table 2), and confidence intervals for AUROC values were calculated via bootstrapping.

**Table 1:** Patient Characteristics (*n* = 336)

	Labels	Appendicitis (*n* = 167)	Other AAP causes (*n* = 169)	*P* value
**ED Intake**				
Age		33 (18.5–51.5)	50 (27.0–68.0)	0.000
Sex	Male	43%	36%	0.274
	Female	57%	64%	
Referrer	Primary Care Physician	74%	57%	0.001
	Self-referral	21%	37%	0.001
	Hospital	3%	2%	0.750
	Ambulance	1%	2%	0.371
	Other Facility	0%	1%	1.000
	Not reported	2%	1%	0.370
Pain Rating		6 (3.5–8.0)	6 (4.0–8.0)	0.227
**Vital Signs**				
Temperature [°C]		37.3 (36.9–37.7)	37.0 (36.6–37.3)	0.000
Mean Arterial Pressure [mmHg]		93 (85–100)	100 (90–109)	0.000
Heart Rate [bpm]		82 (74–96.5)	85 (74–99)	0.730
Saturation [O^2^]		99 (98–100)	98 (96–100)	0.004
**Medical history**				
Development of complaints	Increase	36%	28%	0.375
	Decrease	5%	9%	
	Unaltered	4%	4%	
	Combination	3%	4%	
	Not reported	52%	56%	
Vomiting		56%	51%	0.272
Anorexia		37%	33%	0.515
Fever		19%	11%	0.083
Movement Pain		20%	10%	0.013
Transportation Pain		45%	28%	0.002
Pain Migration	RLQ	36%	5%	0.000
	Flanks	0%	2%	0.122
	Absent	1%	1%	1.000
	Not reported	63%	91%	0.000
Pain Location	Right Lower Quadrant	37%	25%	0.033
	Left Lower Quadrant	0%	2%	0.123
	Right Upper Quadrant	1%	3%	0.448
	Epigastric Region	4%	5%	1.000
	Hypogastric Region	1%	1%	1.000
	Periumbilical	16%	12%	0.344
	Diffuse	8%	7%	0.540
	Combination	26%	30%	0.596
**Physical Examination**				
Pain Location	Right Lower Quadrant	38%	16%	0.000
	Left Lower Quadrant	1%	3%	0.215
	Right Upper Quadrant	1%	3%	0.215
	Epigastric Region	0%	2%	0.248
	Hypogastric Region	0%	1%	1.000
	Periumbilical	4%	3%	0.767
	Diffuse	10%	22%	0.003
	Combination	31%	25%	0.276
	Not Reported	26%	25%	1.000
McBurney’s Sign	Present	58%	15%	0.000
Rebound Tenderness	Present	38%	20%	0.000
**Laboratory Test Results**				
C-Reactive Protein [mg/L]		49.0 (14–118)	20.5 (4–93.5)	0.000
Neutrophils [x10^9/L]		11.9 (± 4.9)	8.8 (± 4.7)	0.000
Leukocytes [x10^9/L]		14.7 (± 5.0)	11.7 (± 5.0)	0.000
Potassium [mmol/L]		3.8 (3.7–4.1)	4.1 (3.7–4.3)	0.001
Monocytes [x10^9/L]		0.7 (0.5–1)	0.6 (0.4–0.8)	0.000
Protein in Urine	+	11%	7%	0.196
	++	4%	4%	
	+++	1%	1%	
	Negative	59%	53%	
	Trace	13%	17%	
	Not Reported	11%	20%	

## Results

### Clinical parameters

Statistical analysis showed that cases with appendicitis exhibited distinct patterns compared to other AAP causes (Table 1). In medical history findings, symptoms such as migration to the right lower quadrant, continuous pain, transportation pain, and movement pain were significantly more prevalent. In physical examination findings, pain in the right lower quadrant, McBurney’s sign, and rebound tenderness were more frequently observed. Laboratory test results for appendicitis showed significantly elevated levels of C-reactive protein, leukocytes, neutrophils, as well as a notably lower potassium concentration.

### Model performance

**Fig. 1:**
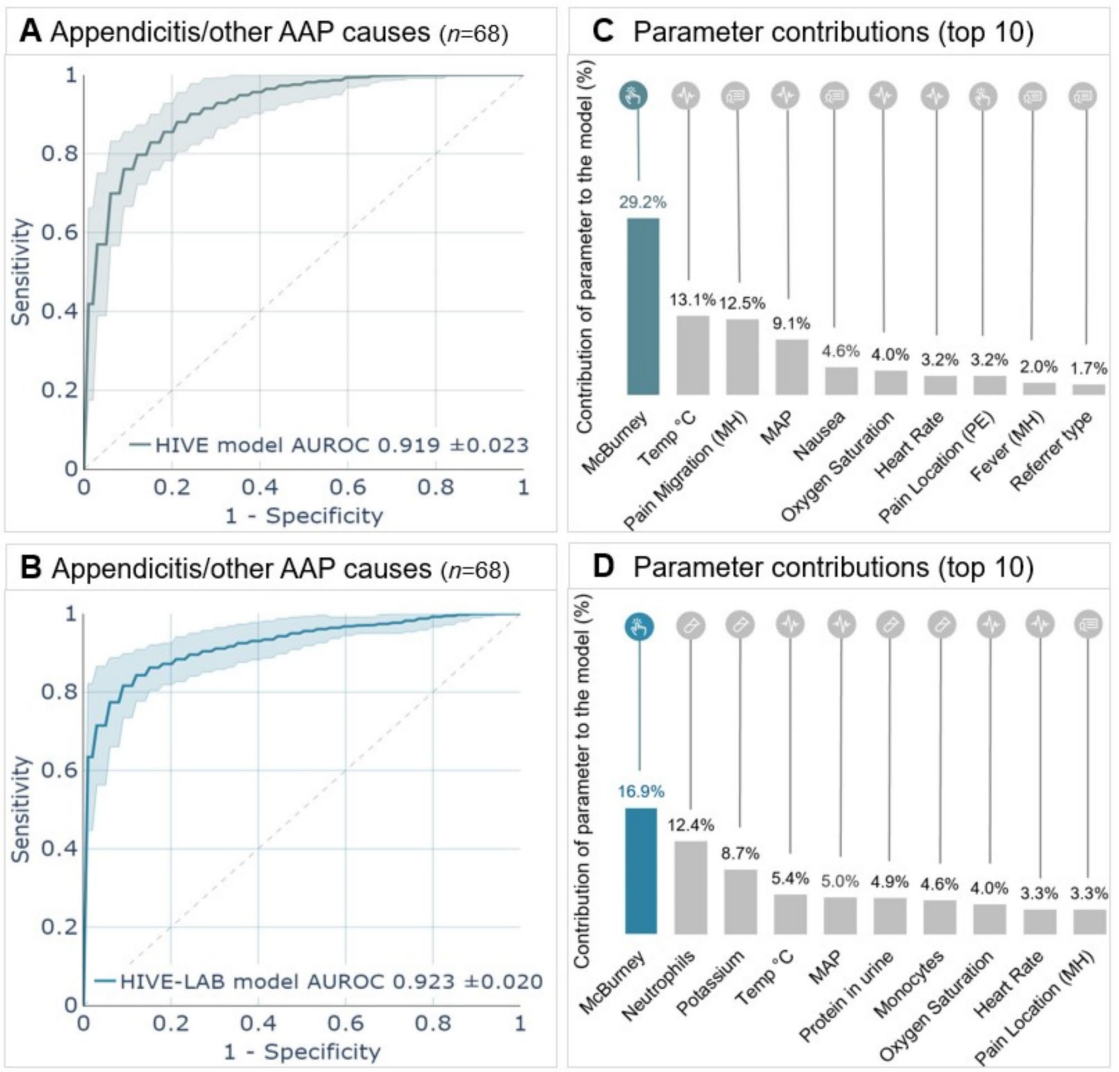
Receiver operating characteristic (ROC) plots for the HIVE model (**A**) and the HIVE-LAB model (**B**) in predicting appendicitis versus other causes of AAP using the validation population. The top 10 individual parameter contributions to the models are represented by SHapley Additive exPlanations (SHAP) values scaled and plotted as percentage contributions to the prediction (**C**,** D**). Parameters outside the top 10 contribute a combined total of 17.5% to the HIVE model and 32.5% to the HIVE-LAB model. HIVE, intake, medical *HI*story, *V*ital signs, physical *E*xamination; HIVE-LAB, intake, medical *HI*story, *V*ital signs, physical *E*xamination, *L*aboratory testing; AAP, acute abdominal pain; Temp, Temperature; MAP, Mean Arterial Pressure; MH, Medical History; PE, Physical Examination

In differentiating appendicitis from other AAP causes, the HIVE model achieved an AUROC of 0.919 (± 0.024) on the validation set (Fig. 1A), while the HIVE-LAB model reached an AUROC of 0.923 (± 0.020) (Fig. 1B), with no statistically significant difference (Table 2). Using SHAP values, the top 10 contributing parameters for each model were identified. For the HIVE model, these parameters in descending order were: (1) McBurney’s sign, (2) body temperature, (3) pain migration, (4) mean arterial pressure (MAP), (5) nausea, (6) oxygen saturation, (7) heart rate, (8) pain location (physical examination), (9) fever (medical history), (10) referrer type (e.g. primary care physician, self-referrer, ambulance) (Fig. 1C). These parameters collectively accounted for 82.5% of the total contribution of all parameters in the HIVE model. For the HIVE-LAB model these were: (1) McBurney’s sign, (2) neutrophils, (3) potassium concentration, (4) body temperature, (5) MAP, (6) protein in urine, (7) monocytes, (8) oxygen saturation, (9) heart rate, (10) pain location (medical history) (Fig. 1D). Despite neutrophils and potassium being highly ranked in the HIVE-LAB model, the overall discriminative ability (AUROC) remained similar to the HIVE model. These 10 parameters accounted for 68.5% of the total contribution of all parameters in the HIVE-LAB model. This indicated that adding laboratory parameters did not provide additional predictive power but instead redistributed the importance among the features. The contribution of all parameters is shown in Supplemental Tables 4 A and 4B in Additional File 1.

**Table 2:** Statistical comparison of AUROC values among ML models, ED physicians, and the Alvarado scoring system using DeLong’s Test

Comparison	AUROC ± CI	*P* value
**ML models**		
HIVE	0.919 ± 0.023	0.978
HIVE-LAB	0.923 ± 0.020	
**HIVE model / ED physicians without lab**		
Physician 1	0.919 ± 0.023 / 0.894 ± 0.076	0.375
Physician 2	0.919 ± 0.023 / 0.826 ± 0.106	0.037
Physician 3	0.919 ± 0.023 / 0.791 ± 0.117	0.007
**HIVE-LAB model / ED physicians with lab**		
Physician 1	0.923 ± 0.020 / 0.923 ± 0.067	0.796
Physician 2	0.923 ± 0.020 / 0.892 ± 0.078	0.353
Physician 3	0.923 ± 0.020 / 0.859 ± 0.098	0.118
**ED physicians without / with lab**		
Physician 1	0.894 ± 0.076 / 0.923 ± 0.067	0.182
Physician 2	0.826 ± 0.106 / 0.892 ± 0.078	0.058
Physician 3	0.791 ± 0.117 / 0.859 ± 0.098	0.177
**ML models / Alvarado**		
HIVE vs. Alvarado	0.919 ± 0.023 / 0.824 ± 0.095	0.033
HIVE-LAB vs. Alvarado	0.923 ± 0.020 / 0.824 ± 0.095	0.031
**ED Physicians without lab / Alvarado**		
Physician 1	0.894 ± 0.076 / 0.824 ± 0.095	0.247
Physician 2	0.826 ± 0.106 / 0.824 ± 0.095	0.980
Physician 3	0.791 ± 0.117 / 0.824 ± 0.095	0.646
**ED Physicians with lab / Alvarado**		
Physician 1	0.923 ± 0.067 / 0.824 ± 0.095	0.071
Physician 2	0.892 ± 0.078 / 0.824 ± 0.095	0.240
Physician 3	0.859 ± 0.098 / 0.824 ± 0.095	0.599

### Comparison of ML models with ED physicians’ diagnostic performance

**Fig. 2:**
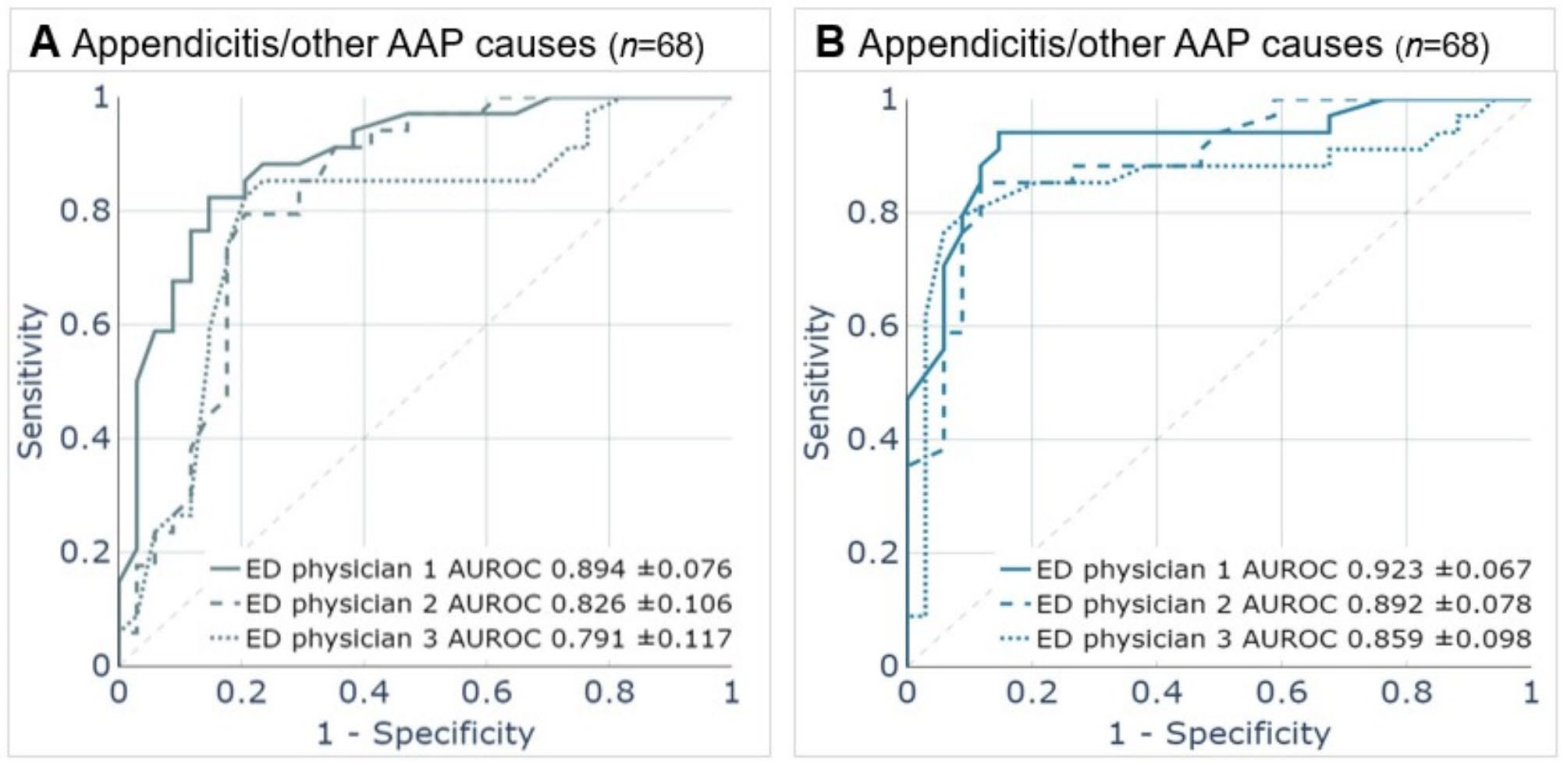
Receiver operating characteristic (ROC) plots illustrating the performance of three ED physicians in diagnosing cases of appendicitis versus other causes of AAP within the same validation population. (**A**) Performance of ED physicians using intake, medical history, vital signs, physical examination information. (**B**) Performance of ED physicians extended with laboratory test results. ED, emergency department; AAP, acute abdominal pain

During their initial assessment of each case in the validation set, reviewing intake information, vital signs, medical history, and physical examination findings, the three ED physicians achieved AUROC scores of 0.894 (± 0.076), 0.826 (± 0.106), and 0.791 (± 0.117) in differentiating appendicitis from other causes of AAP (Fig. 2A). The HIVE model achieved an AUROC of 0.919 (± 0.023) and showed significantly higher performance than two of the three physicians (Fig. 1A; Table 2).

Upon reevaluation, adding laboratory test results did not lead to a statistically significant improvement in the performance of the ED physicians, with AUROCs of 0.923 (± 0.067), 0.892 (± 0.078), and 0.859 (± 0.098) (Fig. 2B). The HIVE-LAB model, with an AUROC of 0.923 (± 0.020), neither showed a significantly different performance compared to the ED physicians in this reevaluation (Fig. 1B; Table 2).

### Evaluation of the Alvarado scoring system and comparison with ML models and ED physicians

**Fig. 3:**
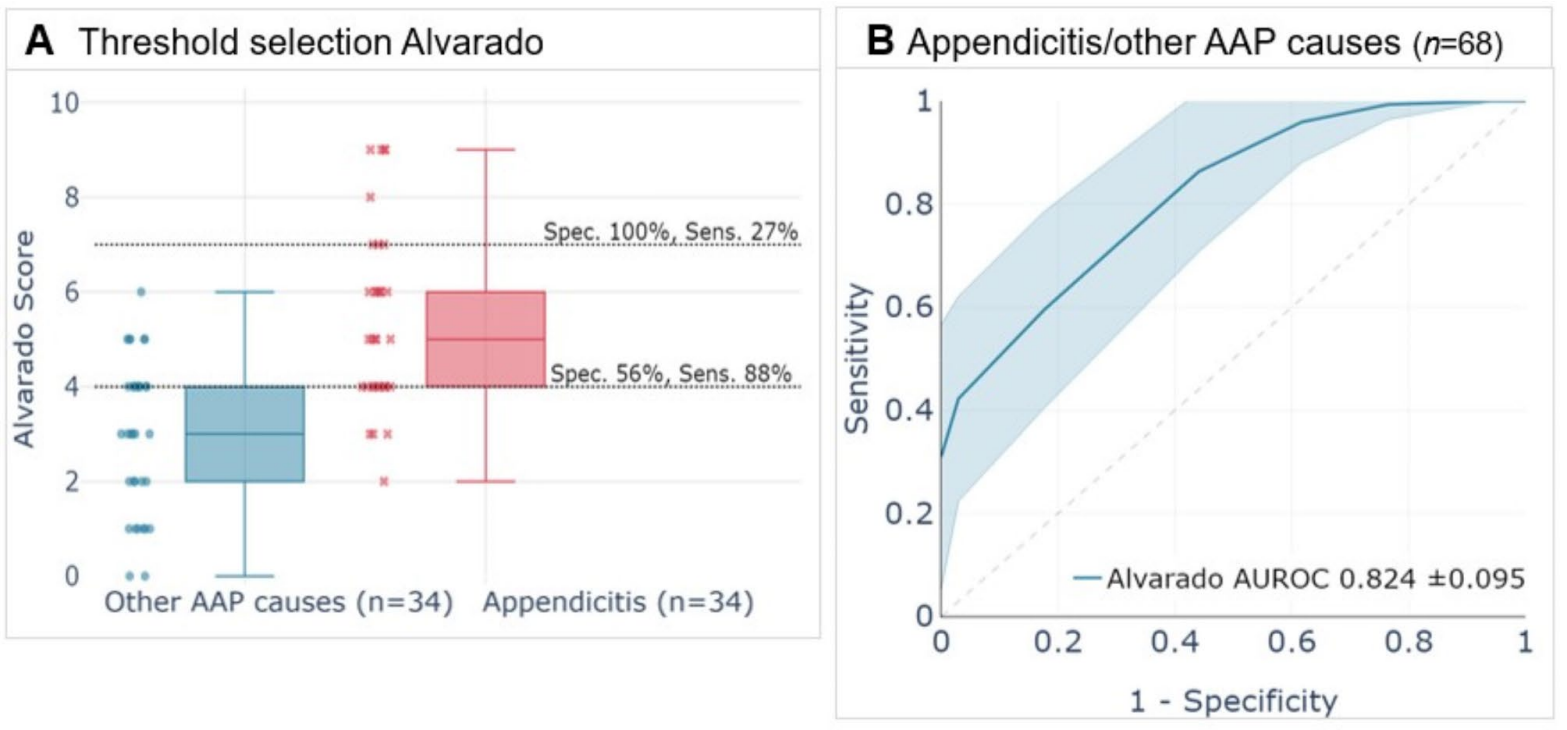
(**A**) Box plots displaying the Alvarado score distributions for cases with appendicitis (*n* = 34) and other causes of AAP (*n* = 34). Sensitivity and specificity thresholds are highlighted for ruling out appendicitis at a score of ≤ 4 (56% specificity, 88% sensitivity) and for identifying appendicitis at a score of ≥ 7 (27% sensitivity, 100% specificity). (**B**) Receiver operating characteristic (ROC) plot using the Alvarado scoring system to predict the risk of appendicitis in the validation population. AAP, acute abdominal pain

The performance of the Alvarado scoring system was evaluated using the same validation set. The first analysis focused on specific sensitivity and specificity thresholds to assess its utility in ruling out and identifying appendicitis (Fig. 3A). At a threshold of ≤ 4, indicative of low risk, the Alvarado scoring system correctly ruled out 56% of patients without appendicitis (specificity of 56%), while 88% of patients with appendicitis had scores higher than 4 (sensitivity of 88%). When the threshold was raised to ≥ 7, indicative of high risk, the Alvarado scoring system correctly identified 27% of patients with appendicitis (sensitivity of 27%) and correctly ruled out 100% of patients without appendicitis (specificity of 100%).

The second analysis focused on the ability of the Alvarado scoring system to distinguish between appendicitis and other AAP causes compared to the ML models and the ED physicians. The Alvarado scoring system achieved a significantly lower AUROC of 0.824 (± 0.095) compared to the HIVE model (0.919 ± 0.023) and the HIVE-LAB model (0.923 ± 0.020) (Figs. 1A-B and 3B; Table 2). The performance of ED physicians was also compared to the Alvarado scoring system, both with and without laboratory test results (Figs. 2A-B and 3B; Table 2). Without laboratory test results, the ED physicians achieved AUROCs of 0.894 (± 0.076), 0.826 (± 0.106), and 0.791 (± 0.117), which were not statistically significantly different from the AUROC of the Alvarado scoring system of 0.824 (± 0.095). Similarly, including laboratory test results did not lead to a statistically significant improvement in the ED physicians’ AUROCs of 0.923 (± 0.067), 0.892 (± 0.078), and 0.859 (± 0.098) compared to the Alvarado scoring system.

## Discussion

Diagnosing patients with AAP is clinically challenging due to the broad spectrum of symptoms and potential underlying conditions. This challenge contributes to a risk of misdiagnosis and to prolonged stays at the ED. Patients suspected of appendicitis are often misdiagnosed, which can result in missed diagnoses or negative appendectomies. This study demonstrates that an ML model using only vital signs, medical history, and physical examination data can accurately diagnose appendicitis early in the ED workup, matching or even surpassing the performance of ED physicians. Notably, incorporating laboratory test results did not significantly enhance the model’s predictive accuracy for diagnosing appendicitis.

These findings have important clinical implications. First, the ML models can assist in clinical decision-making. They are particularly useful when laboratory results are delayed or unavailable. By reducing dependence on laboratory testing or even imaging studies, they optimize resource efficiency. This expedites decision-making, enables earlier treatment, shortens hospital stays, and reduces costs. Second, the ML models and ED physicians outperformed the Alvarado score, showing superior ability to capture complex clinical relationships and rendering the Alvarado score redundant in our setting. Third, variability among ED physicians highlights the influence of individual experience. The ML models provide consistent, objective assessments, standardizing diagnostic accuracy across clinicians. This is valuable in high-pressure ED settings where biases and fatigue may impact decisions, and less experienced physicians (in residence) may benefit from additional support. Even non-significant improvements found in AUROCs between ML models and ED physicians can reduce missed diagnoses and unnecessary surgeries.

Multiple scoring and artificial intelligence (AI)-based systems have been developed to improve diagnostic accuracy for AAP, offering valuable opportunities to enhance the diagnostic workup at the ED or surgery department. ML studies have employed a wide range of algorithms to predict appendicitis [17, 18, 23, 31, 32]. Several studies have successfully benchmarked ML models against the Alvarado score, demonstrating improved diagnostic accuracy [20, 23, 33]. However, these studies exhibit significant heterogeneity in terms of patient populations, input features, ML algorithms, and outcome measures, complicating direct comparison between ML algorithms and models [15]. While existing models have made notable advancements in appendicitis diagnosis, they differ from the design of our study and face certain limitations. They typically require both clinical and laboratory data, or laboratory data alone [10–12, 20–29]. Including laboratory data can act as an additional obstacle in the diagnostic workup and delay diagnosis, while clinical suspicion is often established before laboratory test results are available. Moreover, many ML models are exclusively developed using data from patients who have undergone appendectomies, with the aim to reduce unnecessary surgeries, and were not designed to identify appendicitis in patients entering the ED [16–19, 34]. Additionally, these models have not been directly compared to ED physicians, leaving their potential to complement clinical expertise unclear.

Implementing ML models in clinical practice requires selecting an appropriate threshold that balances specificity and sensitivity, and optimizes positive or negative predictive values (PPV, NPV). This selection depends on local requirements and preferences, related to workload, appendicitis prevalence, and how referral pathways are organized from primary care to the ED (e.g. the ratio of self-referred to those referred by primary care) [5, 35]. Considering these factors, the HIVE model developed in our study enables effective risk stratification of appendicitis in patients with AAP, offering three potential implementation strategies. First, by leveraging a high PPV, the HIVE model can flag patients with a high predicted probability of appendicitis early in the ED workflow. Alternatively, primary care physicians could apply the HIVE model before referring patients to the ED, potentially improving the accuracy of referrals for suspected appendicitis. In both scenarios, patients can be directed immediately to the medical imaging department or surgery department while awaiting imaging, thereby reducing the burden on the ED. Second, by utilizing a high NPV, the HIVE model can identify patients with a low predicted probability of appendicitis. Although this does not rule out other serious causes of AAP, it allows clinicians to consider alternative diagnoses earlier in the evaluation process. For these patients, unnecessary imaging studies specifically targeting appendicitis, as well as negative appendectomies, can be avoided. Third, patients with medium predicted probabilities represent diagnostic uncertainty necessitating careful clinical assessment. The model helps flag these patients, prompting physicians to conduct further evaluations, such as additional diagnostic tests or a period of observation, depending on clinical urgency. This approach ensures that patients who fall into a moderate risk category receive appropriate attention, reducing the likelihood of missed or delayed diagnoses.

However, several limitations must be acknowledged. First, both models rely on the medical history and physical examination notes documented by ED physicians. The models’ performance is inherently tied to the accuracy and completeness of these assessments, including the differential diagnoses considered and the thoroughness of physical examinations. To mitigate potential bias, it is crucial that ED physicians conduct comprehensive assessments and document their findings meticulously. Therefore, ED physicians follow a standardized set of components and questions for medical history and physical examination, ensuring these are completed for every patient regardless of clinical suspicion [36] This standardized approach is integrated into the electronic health record system to promote consistency. Second, some parameters identified as important by the models may not typically be emphasized in diagnosing appendicitis. However, these parameters could be important for other AAP causes or may enhance the models’ overall predictive performance when interpreted in combination with other clinical parameters. Third, both ML models were developed within a single-center context. Deploying them in different settings or patient populations would require a multi-center set-up and external validation to evaluate their generalizability and reliability across diverse clinical environments and patient demographics [37, 38]. Our study was conducted in a Dutch hospital where primary care physicians act as gatekeepers to ED access, leading to a higher prevalence of serious conditions like appendicitis in our cohort. This referral pattern may not represent ED settings without such strong gatekeeping systems or regions with a shortage of primary care physicians. For these settings, calibrating and possibly retraining the models to accommodate more heterogeneous patient populations is recommended. Furthermore, variations in routine laboratory testing protocols and laboratory analyzers used across EDs may affect the applicability of our HIVE-LAB model. In contrast, the HIVE model, which relies only on vital signs and clinical parameters, may offer greater generalizability to different clinical settings. Expanding and validating the HIVE model across diverse patient populations and clinical environments is a promising direction for future research. Fourth, scaling our model to other contexts is currently limited by the manual processing required for unstructured medical history and physical examination data. Future studies should investigate automated methods for processing such unstructured data, which would enable the use of larger and more diverse datasets without necessitating extensive resources. The annotated data from our study could serve as a valuable basis for developing these automated processing techniques. Lastly, another challenge is integrating ML models into clinical workflows. Embedding these tools into electronic health record systems or dashboards with user-friendly interfaces and actionable outputs, based on agreed PPV/NPV thresholds, is essential to streamline the workflow for appendicitis. Training ED and surgical staff to interpret predictions and understand limitations, such as applicability to patients triaged for acute abdominal pain, is essential. Pilot studies or phased rollouts can build trust, address barriers, and ensure surgeon acceptance for broader implementation.

In conclusion, this study highlights the potential of an ML model to aid physicians in diagnosing appendicitis using only vital signs, medical history, and physical examination data, without relying on laboratory testing. The model outperformed the Alvarado scoring system and two out of three experienced ED physicians in a reader study. The comparable performance between the Alvarado score and ED physicians underscores the need for enhanced diagnostic tools. Integrating this model into emergency care can enhance the early differentiation of appendicitis from other causes of AAP during the ED workup.

## Electronic Supplementary Material

Below is the link to the electronic supplementary material.

Supplementary Material 1

Supplementary Material 2

## Data Availability

Data is provided within the manuscript and supplementary information files. Code and models are available via repositories: Github: https://github.com/aschipper/AI-for-Appendicitis Figshare: DOI: 10.6084/m9.figshare.27194622.

## Abbreviations

AAP: Acute Abdominal Pain, ML, machine learning
AI: Artificial Intelligence
ED: Emergency Department
AUROC: Area under the receiver operating curve
XGBoost: eXtreme Gradient Boosting
SHAP: SHapley Additive exPlanations
CT: Computed tomography
ICD-10: International Classification of Diseases 10th Revision
HIVE: History Intake Vitals Examination
HIVE-LAB: History Intake Vitals Examination Laboratory Tests
MANTRELS: Migration, Anorexia, Nausea-vomiting, Tenderness in right lower quadrant, Rebound pain, Elevation of temperature, Leukocytosis, Shift to the left
MAP: Mean arterial pressure
MH: Medical history, PE, physical examination
PPV: Positive predictive values
NPV: Negative predictive values
