# Supplementary material for: Machine-learning based prediction of appendicitis for patients presenting with acute abdominal pain at the emergency department
Source: World J Emerg Surg. 2024 Dec 23;19:40. doi: 10.1186/s13017-024-00570-7 (PMC11664873; doi:10.1186/s13017-024-00570-7)
Supplement: Supplementary file 1 — Supplementary Material 1 [file 13017_2024_570_MOESM1_ESM.docx]

**SUPPLEMENTARY TABLES**

This file contains an overview of the parameters provided as input to the model of the patients in our dataset (Table 1A – 1E). The p values indicate if there is a statistical difference between appendicitis and other AAP causes. In Table 2, an overview of the Alvarado score can be found. In Table 3, the TRIPOD checklist. In Table 4A and 4B, an overview of the parameter contributions to the HIVE and HIVE-LAB models is provided.

**Supplemental Tables**

**Table 1A ED Intake Parameters (*n*=336)**

| **Parameters** | **Labels** | **Appendicitis (*n*=167)** | **Other AAP causes (*n*=169)** | ***P* value** |
| --- | --- | --- | --- | --- |
| **Categorical Parameters** | | | | |
| **Sex** | **Male** | 43% | 36% | 0.274 |
|  | **Female** | 57% | 64% |  |
| **Referred by** | **General Practitioner** | 74% | 57% | 0.001 |
|  | **Self-referral** | 21% | 37% | 0.001 |
|  | **Hospital** | 3% | 2% | 0.750 |
|  | **Ambulance** | 1% | 2% | 0.371 |
|  | **Other Facility** | 0% | 1% | 1.000 |
|  | **Not reported** | 2% | 1% | 0.370 |
| **Transport** | **Ambulance** | 4% | 15% | 0.000 |
|  | **Own Transport** | 96% | 84% | 0.000 |
|  | **Other** | 0% | 1% | 1.000 |
| **Numeric Parameters** | | | | |
| **Number of ED visits in episode** |  | 1  (1 – 1) | 1  (1 – 1) | 0.041 |
| **Age** |  | 33  (18.5 – 51.5) | 50  (27.0 – 68.0) | 0.000 |
| **Pain Rating** |  | 6  (3.5 – 8) | 6  (4 – 8) | 0.227 |

**Table 1B ED Vital Signs (n=336)**

| **Parameters** | **Labels** | **Appendicitis (*n*=167)** | **Other AAP causes (*n*=169)** | ***P* value** |
| --- | --- | --- | --- | --- |
| **Categorical Parameters** | | | | |
| **EMV** |  | 28% | 34% | 0.267 |
| **Q-SOFA** |  | 4% | 6% | 0.728 |
| **Numerical Parameters** | | | | |
| **Diastolic Blood Pressure [mmHg]** |  | 76.5 (± 11.4) | 82.4 (± 13.9) | 0.000 |
| **Systolic Blood Pressure [mmHg]** |  | 127  (117 – 138) | 132  (118.5 – 148) | 0.043 |
| **Mean Arterial Pressure [mmHg]** |  | 93  (85 – 100) | 100  (90 – 109) | 0.000 |
| **Respiratory Rate** |  | 16  (15 – 18) | 17  (14.5 – 20) | 0.244 |
| **Heart Rate [bpm]** |  | 82  (74 – 96.5) | 85  (74 – 99) | 0.730 |
| **SIRS** |  | 0  (0 – 1) | 0  (0 – 1) | 0.176 |
| **Oxygen Saturation** |  | 99  (98 – 100) | 98  (96 – 100) | 0.004 |
| **Temp [°C]** |  | 37.3  (36.9 – 37.7) | 37  (36.6 – 37.3) | 0.000 |

**Table 1C ED Laboratory Parameters (n=336)**

| **Parameters** | | **Labels** | **Analyzer** | **Appendicitis (*n*=167)** | **Other AAP causes (*n*=169)** | ***P* value** |
| --- | --- | --- | --- | --- | --- | --- |
| **Categorical Parameters** | | | | | | |
| **Leukocytes Urine** | | **+** | Roche Miditron; Siemens Advia Clinitek | 14% | 18% | 0.088 |
|  |  | **++** |  | 5% | 9% |  |
|  |  | **+++** |  | 3% | 4% |  |
|  |  | **Negative** |  | 1% | 1% |  |
|  |  | **Trace** |  | 71% | 56% |  |
|  |  | **Not Reported** |  | 8% | 9% |  |
| **Glucose Urine** | | **+** | Roche Miditron; Siemens Advia Clinitek | 11% | 20% | 0.246 |
|  |  | **++** |  | 1% | 0% | 1.000 |
|  |  | **+++** |  | 1% | 1% | 0.123 |
|  |  | **Negative** |  | 0% | 2% | 0.012 |
|  |  | **Trace** |  | 86% | 75% | 0.622 |
|  |  | **Not Reported** |  | 1% | 2% | 0.049 |
| **Nitrites in Urine** | | **Positive** | Roche Miditron; Siemens Advia Clinitek | 11% | 20% | 0.090 |
|  |  | **Negative** |  | 3% | 4% |  |
|  |  | **Not Reported** |  | 86% | 76% |  |
| **Ketones in Urine** | | **+** | Roche Miditron; Siemens Advia Clinitek | 11% | 20% | 0.004 |
|  |  | **++** |  | 13% | 15% |  |
|  |  | **+++** |  | 5% | 5% |  |
|  |  | **Negative** |  | 11% | 2% |  |
|  |  | **Trace** |  | 45% | 49% |  |
|  |  | **Not Reported** |  | 14% | 9% |  |
| **Protein in Urine** | | **+** | Roche Miditron; Siemens Advia Clinitek | 11% | 20% | 0.196 |
|  |  | **++** |  | 11% | 7% |  |
|  |  | **+++** |  | 4% | 4% |  |
|  |  | **Negative** |  | 1% | 1% |  |
|  |  | **Trace** |  | 59% | 53% |  |
|  |  | **Not Reported** |  | 13% | 17% |  |
| **Blood in Urine** | | **+** | Roche Miditron; Siemens Advia Clinitek | 11% | 20% | 0.042 |
|  |  | **++** |  | 11% | 9% |  |
|  |  | **+++** |  | 11% | 5% |  |
|  |  | **Negative** |  | 4% | 8% |  |
|  |  | **Trace** |  | 43% | 43% |  |
|  |  | **Not Reported** |  | 20% | 15% |  |
| **HCG pregnancy test** | | **Negative** | Roche Miditron; Siemens Advia Clinitek | 11% | 20% | 0.416 |
|  |  | **Not Reported** |  | 28% | 24% |  |
| **Numeric Parameters** | | | | | | |
| **Lactate Dehydrogenase [U/L]** |  | | Siemens Dimension Vista; Siemens Advia XPT; Siemens Atellica | 191.6 (± 32.3) | 222.1 (± 93.1) | 0.001 |
| **Neutrophils [x10^9/L]** |  | | Siemens Advia 2120; Sysmex XN-serie | 11.9 (± 4.9) | 8.8 (± 4.7) | 0.000 |
| **Leukocytes [x10^9/L]** |  | | Siemens Advia 2120; Sysmex XN-serie | 14.7 (± 5.0) | 11.7 (± 5.0) | 0.000 |
| **Total Protein [g/L]** |  | | Siemens Dimension Vista; Siemens Advia XPT; Siemens Atellica | 74.0 (± 5.1) | 72.3 (± 5.5) | 0.017 |
| **Calcium [mmol/L]** |  | | Siemens Dimension Vista; Siemens Advia XPT; Siemens Atellica | 2.4 (± 0.1) | 2.4 (± 0.1) | 0.894 |
| **C-Reactive Protein [mg/L]** |  | | Siemens Dimension Vista; Siemens Advia XPT; Siemens Atellica | 49  (14 – 118) | 20.5  (4 – 93.5) | 0.000 |
| **Hemoglobin [mmol/L]** |  | | Siemens Advia 2120; Sysmex XN-serie | 8.7  (8.0 – 9.2) | 8.6  (8.1 – 9.0) | 0.645 |
| **Mean Corpuscular Volume [fL]** |  | | Siemens Advia 2120; Sysmex XN-serie | 87  (84 – 90) | 89  (85 – 91) | 0.005 |
| **Red Blood Cells [x10^12/L]** |  | | Siemens Advia 2120; Sysmex XN-serie | 4.7  (4.4 – 5.0) | 4.7  (4.3 – 5.0) | 0.331 |
| **Platelets [x10^9/L]** |  | | Siemens Advia 2120; Sysmex XN-serie | 269  (228 – 329) | 280  (222 – 338) | 0.293 |
| **Hematocrit [L/L]** |  | | Siemens Advia 2120; Sysmex XN-serie | 0.4  (0.4 – 0.4) | 0.4  (0.4 – 0.4) | 0.498 |
| **Creatinine [µmol/L]** |  | | Siemens Dimension Vista; Siemens Advia XPT; Siemens Atellica | 64.5  (54.3 – 77.8) | 66  (55 – 83) | 0.123 |
| **Urea [mmol/L]** |  | | Siemens Dimension Vista; Siemens Advia XPT; Siemens Atellica | 4.2  (3.5 – 5.3) | 4.7  (3.8 – 7.0) | 0.003 |
| **Total Bilirubin [µmol/L]** |  | | Siemens Dimension Vista; Siemens Advia XPT; Siemens Atellica | 11  (7 – 15) | 9  (7 – 15) | 0.369 |
| **Direct Bilirubin[µmol/l]** |  | | Siemens Dimension Vista; Siemens Advia XPT; Siemens Atellica | 4  (2 – 5) | 3  (2 – 5) | 0.342 |
| **ALAT [U/L]** |  | | Siemens Dimension Vista; Siemens Advia XPT; Siemens Atellica | 18  (13 – 25.8) | 19  (14 – 32.5) | 0.082 |
| **ASAT [U/L]** |  | | Siemens Dimension Vista; Siemens Advia XPT; Siemens Atellica | 18  (14 – 22) | 20  (16 – 30) | 0.002 |
| **Alkaline Phosphatase [U/L]** |  | | Siemens Dimension Vista; Siemens Advia XPT; Siemens Atellica | 81  (68 – 108.5) | 82  (65 – 107.8) | 0.005 |
| **Gamma-Glutamyl [U/L]** |  | | Siemens Dimension Vista; Siemens Advia XPT; Siemens Atellica | 20  (12 – 32.8) | 21.5  (13 – 39.8) | 0.216 |
| **Lipase [U/L]** |  | | Siemens Dimension Vista; Siemens Advia XPT; Siemens Atellica | 32  (27 – 47) | 33  (26 – 37.8) | 0.179 |
| **Sodium [mmol/L]** |  | | Siemens Dimension Vista; Siemens Advia XPT; Siemens Atellica | 138  (136 – 139) | 138  (136 – 140) | 0.742 |
| **Potassium [mmol/L]** |  | | Siemens Dimension Vista; Siemens Advia XPT; Siemens Atellica | 3.8  (3.7 – 4.1) | 4.1  (3.7 – 4.3) | 0.001 |
| **Glucose [mmol/L]** |  | | Siemens Dimension Vista; Siemens Advia XPT; Siemens Atellica | 5.8  (5.2 – 6.6) | 5.9  (5.2 – 7.8) | 0.029 |
| **Chloride [mmol/L]** |  | | Siemens Dimension Vista; Siemens Advia XPT; Siemens Atellica | 102  (101 – 104) | 104  (99 – 106) | 0.178 |
| **estimated Glomerular Filtration Rate [mL/min/1.73m2]** |  | | Siemens Dimension Vista; Siemens Advia XPT; Siemens Atellica | 90  (90 – 90) | 90  (68 – 90) | 0.000 |
| **Creatine Kinase [U/L]** |  | | Siemens Dimension Vista; Siemens Advia XPT; Siemens Atellica | 75  (51 – 108) | 75  (46 – 113) | 0.999 |
| **Albumin [g/L]** |  | | Siemens Dimension Vista; Siemens Advia XPT; Siemens Atellica | 40  (37 – 42) | 39  (34 – 42) | 0.025 |
| **Eosinophils [x10^9/L]** |  | | Siemens Advia 2120; Sysmex XN-serie | 0.1  (0 – 0.125) | 0.1  (0 – 0.1) | 0.334 |
| **Lymphocytes [x10^9/L]** |  | | Siemens Advia 2120; Sysmex XN-serie | 1.6  (1.1 – 2.3) | 1.6  (1.1 – 2.2) | 0.282 |
| **Basophils [x10^9/L]** |  | | Siemens Advia 2120; Sysmex XN-serie | 0  (0 – 0.1) | 0  (0 – 0.075) | 0.073 |
| **Monocytes [x10^9/L]** |  | | Siemens Advia 2120; Sysmex XN-serie | 0.7  (0.5 – 1) | 0.6  (0.4 – 0.8) | 0.000 |
| **Urine pH** |  | | Roche Miditron; Siemens Advia Clinitek | 6  (6 – 7) | 6  (6 – 7) | 0.674 |

**Table 1D ED Medical History Parameters (*n*=336)**

| **Parameters** | **Label** | **Appendicitis (*n*=167)** | **Other AAP causes (*n*=169)** | ***P* value** |
| --- | --- | --- | --- | --- |
| **Categorical Parameters** | | | | |
| **Development of Complaints** | **Increase** | 36% | 28% | 0.357 |
|  | **Decrease** | 5% | 9% |  |
|  | **Combination** | 3% | 4% |  |
|  | **Unaltered** | 4% | 4% |  |
|  | **Not Reported** | 52% | 56% |  |
| **Dyspnea** |  | 2% | 3% | 0.723 |
| **Pain Migration** | **Flanks** | 0% | 2% | 0.122 |
|  | **Right Lower Quadrant** | 36% | 5% | 0.000 |
|  | **Absent** | 1% | 1% | 1.000 |
|  | **Not Reported** | 63% | 91% | 0.000 |
| **Onset of Pain** | **Acute** | 13% | 20% | 0.107 |
|  | **Gradual** | 2% | 2% | 1.000 |
|  | **Not Reported** | 85% | 78% | 0.093 |
| **Stool Color** | **Different** | 3% | 5% | 0.426 |
|  | **Normal** | 97% | 95% |  |
| **Lung Abnormalities** | **Absent** | 100% | 100% |  |
| **Menstrual Abnormalities** |  | 1% | 1% | 1.000 |
| **Visit Abroad** |  | 2% | 1% | 0.369 |
| **Stool Melaena** |  | 0% | 1% | 1.000 |
| **Anorexia** | **Decrease** | 37% | 33% | 0.515 |
|  | **Equivalent** | 63% | 67% |  |
| **Malaise Complaints** |  | 19% | 17% | 0.847 |
| **Birth Control** |  | 10% | 12% | 0.756 |
| **Last Stool** | **<=2 days** | 49% | 42% | 0.032 |
|  | **>2 days** | 4% | 12% |  |
|  | **Not Reported** | 47% | 46% |  |
| **Sweatiness** |  | 5% | 5% | 0.980 |
| **Pain Manifestation** | **Attack-wise** | 8% | 16% | 0.002 |
|  | **Continuous** | 39% | 24% |  |
|  | **Combination** | 6% | 13% |  |
|  | **Not Reported** | 47% | 47% |  |
| **Swollen Abdomen** |  | 2% | 6% | 0.094 |
| **Urine Color** | **Different** | 3% | 5% | 0.426 |
|  | **Normal** | 97% | 95% |  |
| **Nausea** |  | 56% | 49% | 0.272 |
| **Cold Shivers** |  | 12% | 9% | 0.452 |
| **Hematuria** |  | 1% | 3% | 0.448 |
| **Dysuria** |  | 1% | 6% | 0.041 |
| **Nature of Pain** | **Aching** | 8% | 7% | 0.688 |
|  | **Cramps** | 8% | 7% | 0.838 |
|  | **Stabbing** | 14% | 13% | 0.874 |
|  | **Burning** | 0% | 1% | 1.000 |
|  | **Combination** | 8% | 5% | 0.538 |
|  | **Not Reported** | 62% | 67% | 0.363 |
| **Cardial Abnormalities** | **Absent** | 100% | 100% |  |
| **Pain After Fatty Meal** |  | 1% | 1% | 0.622 |
| **Thoracic Pain** |  | 0% | 2% | 0.123 |
| **Stool Mucus** |  | 2% | 2% | 1.000 |
| **Stoma** |  | 1% | 4% | 0.121 |
| **Stool Consistency** | **Diarrhea** | 11% | 12% | 0.206 |
|  | **Loose** | 20% | 12% |  |
|  | **Normal** | 39% | 37% |  |
|  | **Changing** | 1% | 1% |  |
|  | **Stiff/Obstipation** | 3% | 7% |  |
|  | **Not Reported** | 26% | 31% |  |
| **Movement Urge** |  | 7% | 12% | 0.102 |
| **Coughing** |  | 1% | 3% | 0.448 |
| **Pregnancy** |  | 2% | 1% | 0.369 |
| **Radiating Pain** |  | 10% | 14% | 0.326 |
| **Pollakiuria** |  | 5% | 3% | 0.557 |
| **Surrounding people's complaints** |  | 2% | 0% | 0.122 |
| **Vomiting** |  | 40% | 35% | 0.382 |
| **Leukorrhea** | **Different** | 1% | 1% | 1.000 |
|  | **Normal** | 11% | 8% | 0.365 |
|  | **Not Reported** | 88% | 91% | 0.485 |
| **Hematemesis** |  | 1% | 1% | 1.000 |
| **Transportation Pain** |  | 45% | 28% | 0.002 |
| **Pain Location (AN)** | **Right Lower Quadrant** | 37% | 25% | 0.033 |
|  | **Left Lower Quadrant** | 0% | 2% | 0.123 |
|  | **Right Upper Quadrant** | 1% | 3% | 0.448 |
|  | **Epigastric Region** | 4% | 5% | 1.000 |
|  | **Hypogastric Region** | 1% | 1% | 1.000 |
|  | **Periumbilical** | 16% | 12% | 0.344 |
|  | **Diffuse** | 8% | 7% | 0.540 |
|  | **Combination** | 26% | 30% | 0.596 |
|  | **No Pain Flanks** | 0% | 1% | 1.000 |
|  | **Not Reported** | 7% | 15% | 0.035 |
| **Short of Breath** |  | 2% | 3% | 0.072 |
| **Strangury** |  | 5% | 5% | 1.000 |
| **STD-risk** |  | 0% | 1% | 1.000 |
| **Fever** |  | 19% | 11% | 0.083 |
| **Prior Episode** |  | 5% | 13% | 0.026 |
| **Movement Pain** |  | 20% | 10% | 0.013 |
| **Blood in Stool** |  | 2% | 2% | 1.000 |
| **Pain Respiration** |  | 0% | 1% | 1.000 |
| **POCT CRP** |  | 4% | 1% | 0.171 |
| **Food-related** |  | 8% | 8% | 1.000 |
| **Family History** |  | 1% | 4% | 0.182 |
| **Duration Complaints** | **<24h** | 34% | 32% | 0.728 |
|  | **12h-48h** | 28% | 17% | 0.013 |
|  | **>48h-8d** | 11% | 15% | 0.258 |
|  | **>7d-1m** | 6% | 5% | 0.637 |
|  | **>1m** | 2% | 4% | 0.336 |
|  | **Unclear** | 19% | 18% | 0.986 |
|  | **Not Reported** | 1% | 9% | 0.000 |

**Table 1E ED Physical Examination Parameters (*n*=336)**

| **Parameters** | **Labels** | **Appendicitis (*n*=167)** | **Other AAP causes (*n*=169)** | ***P* value** |
| --- | --- | --- | --- | --- |
| **Categorical Parameters** | | | | |
| **Muscular Defense** |  | 14% | 11% | 0.592 |
| **Pain Location (PE)** | **Right Lower Quadrant** | 38% | 16% | 0.000 |
|  | **Left Lower Quadrant** | 1% | 3% | 0.215 |
|  | **Right Upper Quadrant** | 1% | 3% | 0.215 |
|  | **Epigastric Region** | 0% | 2% | 0.248 |
|  | **Hypogastric Region** | 0% | 1% | 1.000 |
|  | **Periumbilical** | 4% | 3% | 0.767 |
|  | **Diffuse** | 10% | 22% | 0.003 |
|  | **Combination** | 31% | 25% | 0.276 |
|  | **Not Reported** | 26% | 25% | 1.000 |
| **Psoas Sign** |  | 8% | 4% | 0.238 |
| **Inguinal Abnormalities** | **Swelling** | 1% | 1% | 1.000 |
|  | **Absent** | 99% | 99% |  |
| **Inguinal Hernia** | **Umbilical** | 1% | 1% | 1.000 |
|  | **Absent** | 99% | 99% |  |
| **Pyrosis** |  | 1% | 2% | 0.685 |
| **Percussion Abdomen** | **Hypertympanic** | 1% | 5% | 0.061 |
|  | **Variable Tympanic** | 61% | 53% | 0.154 |
|  | **Diminished** | 1% | 1% | 1.000 |
|  | **Combination** | 0% | 1% | 0.498 |
|  | **Not Reported** | 37% | 39% | 0.737 |
| **Peritoneal Irritation** |  | 1% | 3% | 0.448 |
| **Percussion Pain** | **Present** | 10% | 5% | 0.151 |
|  | **Absent** | 1% | 1% | 1.000 |
|  | **Not Reported** | 89% | 93% | 0.178 |
| **Palpation Tenderness** |  | 96% | 83% | 0.144 |
| **Murphy’s Sign** |  | 2% | 5% | 0.389 |
| **Rebound Tenderness** |  | 38% | 20% | 0.000 |
| **Pulmonary Abnormal Sounds** | **Crackles** | 1% | 3% | 0.215 |
|  | **Absent** | 99% | 97% |  |
| **Contralateral Rebound Tenderness** |  | 10% | 5% | 0.201 |
| **Character of Abdominal Auscultation** | **Sink-like rumbling** | 1% | 1% | 1.000 |
|  | **High-pitched** | 0% | 2% | 0.123 |
|  | **Combination** | 0% | 1% | 0.498 |
|  | **Normal Sounds** | 4% | 4% | 1.000 |
|  | **Not Reported** | 96% | 92% | 0.249 |
| **Presence of Abdominal Auscultation** | **Hyperactive Peristalsis** | 11% | 9% | 0.474 |
|  | **Normal Peristalsis** | 52% | 41% | 0.049 |
|  | **Scant Peristalsis** | 16% | 21% | 0.258 |
|  | **Absent Peristalsis** | 1% | 4% | 0.067 |
|  | **Combination** | 2% | 2% | 1.000 |
|  | **Not Reported** | 19% | 23% | 0.348 |
| **Costovertebral Angle Tenderness** | **Left** | 1% | 1% | 1.000 |
|  | **Right** | 1% | 4% | 0.067 |
|  | **Absent** | 99% | 95% | 0.611 |
| **Abdomen Palpation Abnormal** |  | 2% | 3% | 1.000 |
| **McBurney** |  | 58% | 15% | 0.000 |
| **Abdominal Inspection** | **Adipose** | 10% | 13% | 0.496 |
|  | **Scars** | 2% | 7% | 0.031 |
|  | **Distended Abdomen** | 3% | 8% | 0.056 |
|  | **Combination** | 2% | 4% | 0.502 |
|  | **No Abnormalities** | 19% | 20% | 1.000 |
|  | **Not Reported** | 64% | 49% | 0.004 |
| **General Impression** | **Not sick, no pain** | 72% | 62% | 0.050 |
|  | **Moderately sick, no pain** | 7% | 7% | 0.832 |
|  | **Moderately sick, moderate pain** | 0% | 1% | 1.000 |
|  | **Moderately sick, with pain** | 0% | 1% | 1.000 |
|  | **Not sick, moderate pain** | 2% | 1% | 0.447 |
|  | **Not sick, with pain** | 2% | 7% | 0.110 |
|  | **Sick, no pain** | 2% | 5% | 0.257 |
|  | **Not Reported** | 14% | 18% | 0.370 |

**Table 2 Alvarado Score**

| **Alvarado Components** | **Score Points** |
| --- | --- |
| **Symptoms** | |
| **Migratory Pain to Right Lower Quadrant** | 1 |
| **Anorexia** | 1 |
| **Nausea or Vomiting** | 1 |
| **Signs** | |
| **Tenderness of the Right Lower Quadrant** | 2 |
| **Rebound Tenderness** | 1 |
| **Temperature >37.3 °C** | 1 |
| **Laboratory** | |
| **Leukocytes Above 10 [x10^9/L]** | 2 |
| **Neutrophilic Count >75 % (Shift to the left)** | 1 |

Alvarado Risk Stratification: Score 1–4 For Low Probability, score 5–6 For Intermediate Probability, score 7–10 For High Probability of Appendicitis.

**Table 3** TRIPOD Checklist: Prediction Model Development


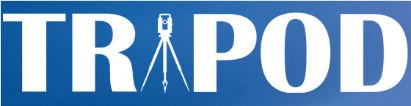


| **Section/Topic** | **Item** | **Checklist Item** | **Page** |
| --- | --- | --- | --- |
| **Title and abstract** |  |  |  |
| Title | 1 | Identify the study as developing and/or validating a multivariable prediction model, the target population, and the outcome to be predicted. | 1 |
| Abstract | 2 | Provide a summary of objectives, study design, setting, participants, sample size, predictors, outcome, statistical analysis, results, and conclusions. | 3 |
| **Introduction** |  |  |  |
| Background and objectives | 3a | Explain the medical context (including whether diagnostic or prognostic) and rationale for developing or validating the multivariable prediction model, including references to existing models. | 4 |
|  | 3b | Specify the objectives, including whether the study describes the development or validation of the model or both. | 5 |
| **Methods** |  |  |  |
| Source of data | 4a | Describe the study design or source of data (e.g., randomized trial, cohort, or registry data), separately for the development and validation data sets, if applicable. | 6 |
|  | 4b | Specify the key study dates, including start of accrual; end of accrual; and, if applicable, end of follow-up. | 6 |
| Participants | 5a | Specify key elements of the study setting (e.g., primary care, secondary care, general population) including number and location of centres. | 6 |
|  | 5b | Describe eligibility criteria for participants. | 6 |
|  | 5c | Give details of treatments received, if relevant. | 6 |
| Outcome | 6a | Clearly define the outcome that is predicted by the prediction model, including how and when assessed. | 6-7, Fig 2B |
|  | 6b | Report any actions to blind assessment of the outcome to be predicted. | 8 |
| Predictors | 7a | Clearly define all predictors used in developing or validating the multivariable prediction model, including how and when they were measured. | 7, Sup Table 1 |
|  | 7b | Report any actions to blind assessment of predictors for the outcome and other predictors. | 8 |
| Sample size | 8 | Explain how the study size was arrived at. | 6 |
| Missing data | 9 | Describe how missing data were handled (e.g., complete-case analysis, single imputation, multiple imputation) with details of any imputation method. | 6-7 |
| Statistical analysis methods | 10a | Describe how predictors were handled in the analyses. | 8-9 |
|  | 10b | Specify type of model, all model-building procedures (including any predictor selection), and method for internal validation. | 8 |
|  | 10d | Specify all measures used to assess model performance and, if relevant, to compare multiple models. | 8-9 |
| Risk groups | 11 | Provide details on how risk groups were created, if done. |  |
| **Results** |  |  |  |
| Participants | 13a | Describe the flow of participants through the study, including the number of participants with and without the outcome and, if applicable, a summary of the follow-up time. A diagram may be helpful. | 6, Fig 2 |
|  | 13b | Describe the characteristics of the participants (basic demographics, clinical features, available predictors), including the number of participants with missing data for predictors and outcome. | 11, Table 1 |
| Model development | 14a | Specify the number of participants and outcome events in each analysis. | 11, Fig 3 |
|  | 14b | If done, report the unadjusted association between each candidate predictor and outcome. | Sup Table 1 |
| Model  specification | 15a | Present the full prediction model to allow predictions for individuals (i.e., all regression coefficients, and model intercept or baseline survival at a given time point). | Github? |
|  | 15b | Explain how to the use the prediction model. | 11 |
| Model performance | 16 | Report performance measures (with CIs) for the prediction model. | 11, Fig 3 |
| **Discussion** |  |  |  |
| Limitations | 18 | Discuss any limitations of the study (such as nonrepresentative sample, few events per predictor, missing data). | 13-14 |
| Interpretation | 19b | Give an overall interpretation of the results, considering objectives, limitations, and results from similar studies, and other relevant evidence. | 13-14 |
| Implications | 20 | Discuss the potential clinical use of the model and implications for future research. | 14 |
| **Other information** |  |  |  |
| Supplementary information | 21 | Provide information about the availability of supplementary resources, such as study protocol, Web calculator, and data sets. | 15 |
| Funding | 22 | Give the source of funding and the role of the funders for the present study. | 15 |

We recommend using the TRIPOD Checklist in conjunction with the TRIPOD Explanation and Elaboration document.

**Table 4A** Parameter contributions HIVE model

| **Parameter** | **Contribution to the ML model** |
| --- | --- |
| **McBurney's Sign** | 29.2% |
| **Temperature [°C]** | 13.1% |
| **Pain Migration (MH)*** | 12.5% |
| **Mean Arterial Pressure [mmHg]** | 9.1% |
| **Nausea** | 4.6% |
| **Oxygen Saturation** | 4.0% |
| **Heart Rate [bpm]** | 3.2% |
| **Pain Location (PE)*** | 3.2% |
| **Fever** | 2.0% |
| **Referrer** | 1.7% |
| **Pain rating** | 1.6% |
| **Pain location (MH)*** | 1.6% |
| **Pain manifestation** | 1.5% |
| **Sex** | 1.3% |
| **Rebound tenderness** | 0.8% |
| **Age [y]** | 0.8% |
| **Anorexia** | 0.7% |
| **Abdominal inspection** | 0.7% |
| **Stool consistency** | 0.6% |
| **Nature of pain** | 0.6% |
| **Onset of pain** | 0.6% |
| **Transport** | 0.5% |
| **Diastolic blood pressure [mmHg]** | 0.4% |
| **Development of complaints** | 0.4% |
| **Abdomen tenderness** | 0.4% |
| **Q-SOFA** | 0.4% |
| **Pollakiuria** | 0.4% |
| **Vomiting** | 0.4% |
| **Food-related** | 0.3% |
| **Percussion pain** | 0.3% |
| **Movement urge** | 0.3% |
| **Last stool** | 0.3% |
| **Transportation pain** | 0.3% |
| **SIRS** | 0.2% |
| **Palpation tenderness** | 0.2% |
| **Duration complaints** | 0.2% |
| **Medical history: appendectomy** | 0.2% |
| **Menstrual abnormalities** | 0.2% |
| **Sweatiness** | 0.2% |
| **Surrounding people's complaints** | 0.2% |
| **Presence of abdominal auscultation** | 0.2% |
| **Hematemesis** | 0.2% |
| **Cold shivers** | 0.2% |
| **Systolic blood pressure [mmHg]** | 0.2% |
| **Thoracic pain** | 0.2% |
| **Swollen abdomen (MH)** | 0.1% |
| **Number of ED visits in episode** | 0.0% |
| **Muscular defense** | 0.0% |
| **EMV** | 0.0% |
| **Respiratory rate** | 0.0% |
| **Short of breath** | 0.0% |
| **Stool color** | 0.0% |
| **Psoas sign** | 0.0% |
| **Lung abnormalities** | 0.0% |
| **Inguinal abnormalities** | 0.0% |
| **Inguinal hernia** | 0.0% |
| **Visit abroad** | 0.0% |
| **Stool melaena** | 0.0% |
| **Pyrosis** | 0.0% |
| **Malaise complaints** | 0.0% |
| **Birth control** | 0.0% |
| **Percussion abdomen** | 0.0% |
| **Peritoneal irritation** | 0.0% |
| **Urine color** | 0.0% |
| **Hematuria** | 0.0% |
| **Dysuria** | 0.0% |
| **Heart abnormalities** | 0.0% |
| **Pain after fatty meal** | 0.0% |
| **Stool mucus** | 0.0% |
| **Stoma** | 0.0% |
| **Coughing** | 0.0% |
| **Murphy's sign** | 0.0% |
| **Pregnancy** | 0.0% |
| **Radiating pain** | 0.0% |
| **Vaginal discharge** | 0.0% |
| **Pulmonary abnormal sounds** | 0.0% |
| **Contralateral rebound tenderness** | 0.0% |
| **Strangury** | 0.0% |
| **STD-risk** | 0.0% |
| **Character of abdominal auscultation** | 0.0% |
| **Costovertebral angle tenderness** | 0.0% |
| **Prior episode** | 0.0% |
| **Movement pain** | 0.0% |
| **Abdomen palpation abnormal** | 0.0% |
| **Blood in stool** | 0.0% |
| **Family history** | 0.0% |
| **Respiration pain** | 0.0% |
| **POCT CRP** | 0.0% |
| **General impression** | 0.0% |

* MH, Medical History; PE, Physical Examination

**Table 4B** Parameter contributions HIVE-LAB model

| **Parameter** | **Contribution to the ML model** |
| --- | --- |
| **McBurney's Sign** | 16.9% |
| **Neutrophils [x10^9/L]** | 12.4% |
| **Potassium [mmol/L]** | 8.7% |
| **Temperature [°C]** | 5.4% |
| **Mean Arterial Pressure [mmHg]** | 5.0% |
| **Protein in Urine** | 4.9% |
| **Monocytes [x10^9/L]** | 4.6% |
| **Oxygen Saturation** | 4.0% |
| **Heart Rate [bpm]** | 3.3% |
| **Pain Location (PE)** | 3.3% |
| **Age [y]** | 3.2% |
| **Pain Migration** | 3.2% |
| **C-Reactive Protein [mg/L]** | 2.4% |
| **Creatine Kinase [U/L]** | 2.3% |
| **estimated Glomerular Filtration Rate [mL/min/1.73m2]** | 1.8% |
| **Pain Location (PE)** | 1.0% |
| **Pain rating** | 0.9% |
| **Hematuria** | 0.9% |
| **Nausea** | 0.9% |
| **Sex** | 0.8% |
| **Ketones in urine** | 0.8% |
| **Leukocytes [x10^9/L]** | 0.8% |
| **Diastolic blood pressure [mmHg]** | 0.8% |
| **Stool consistency** | 0.8% |
| **calcium** | 0.8% |
| **ASAT [U/L]** | 0.7% |
| **Pain manifestation** | 0.7% |
| **Leukocytes Urine** | 0.7% |
| **Nature of pain** | 0.6% |
| **Alkaline Phosphatase [U/L]** | 0.6% |
| **Vomiting** | 0.5% |
| **Creatinine [µmol/L]** | 0.5% |
| **Urea [mmol/L]** | 0.5% |
| **Total Bilirubin [µmol/L]** | 0.5% |
| **Glucose [mmol/L]** | 0.5% |
| **Referrer** | 0.5% |
| **Lactate Dehydrogenase [U/L]** | 0.5% |
| **Abdomen palpation abnormal** | 0.4% |
| **Transport** | 0.4% |
| **Malaise complaints** | 0.4% |
| **Family History** | 0.4% |
| **Lipase [U/L]** | 0.4% |
| **Last stool** | 0.4% |
| **Abdominal inspection** | 0.4% |
| **Anorexia** | 0.3% |
| **Food-related** | 0.2% |
| **Number of ED visits in episode** | 0.0% |
| **Muscular defense** | 0.0% |
| **Systolic blood pressure [mmHg]** | 0.0% |
| **EMV** | 0.0% |
| **SIRS** | 0.0% |
| **Respiratory rate** | 0.0% |
| **Q-SOFA** | 0.0% |
| **Developments of complaints** | 0.0% |
| **Shortness of breath** | 0.0% |
| **Onset of pain** | 0.0% |
| **Stool color** | 0.0% |
| **Psoas sign** | 0.0% |
| **Lung abnormalities** | 0.0% |
| **Menstrual abnormalities** | 0.0% |
| **Inguinal abnormalities** | 0.0% |
| **Inguinal hernia** | 0.0% |
| **Visit abroad** | 0.0% |
| **Medical history: appendectomy** | 0.0% |
| **Stool melaena** | 0.0% |
| **Pyrosis** | 0.0% |
| **Birth control** | 0.0% |
| **Percussion abdomen** | 0.0% |
| **Sweatiness** | 0.0% |
| **Swollen abdomen (MH)** | 0.0% |
| **Peritoneal irritation** | 0.0% |
| **Urine color** | 0.0% |
| **Cold shivers** | 0.0% |
| **Dysuria** | 0.0% |
| **Percussion pain** | 0.0% |
| **Heart abnormalities** | 0.0% |
| **Pain after fatty meal** | 0.0% |
| **Thoracic pain** | 0.0% |
| **Stool mucus** | 0.0% |
| **Stoma** | 0.0% |
| **Movement urge** | 0.0% |
| **Coughing** | 0.0% |
| **Abdomen tenderness** | 0.0% |
| **Murphy's sign** | 0.0% |
| **Rebound tenderness** | 0.0% |
| **Pregnancy** | 0.0% |
| **Radiating pain** | 0.0% |
| **Pollakiuria** | 0.0% |
| **Surrounding people's complaints** | 0.0% |
| **Vaginal discharge** | 0.0% |
| **Hematemesis** | 0.0% |
| **Transportation pain** | 0.0% |
| **Pulmonary abnormal sounds** | 0.0% |
| **Contralateral rebound tenderness** | 0.0% |
| **Strangury** | 0.0% |
| **STD-risk** | 0.0% |
| **Fever** | 0.0% |
| **Character of abdominal auscultation** | 0.0% |
| **Costovertebral angle tenderness** | 0.0% |
| **Prior episode** | 0.0% |
| **Movement pain** | 0.0% |
| **Blood in stool** | 0.0% |
| **Palpation tenderness** | 0.0% |
| **Presence of abdominal auscultation** | 0.0% |
| **Respiration pain** | 0.0% |
| **POCT CRP** | 0.0% |
| **Duration complaints** | 0.0% |
| **General impression** | 0.0% |
| **Hemoglobin [mmol/L]** | 0.0% |
| **Mean Corpuscular Volume [fL]** | 0.0% |
| **Red Blood Cells [x10^12/L]** | 0.0% |
| **Platelets [x10^9/L]** | 0.0% |
| **Hematocrit [L/L]** | 0.0% |
| **Direct Bilirubin[µmol/l]** | 0.0% |
| **ALAT [U/L]** | 0.0% |
| **Gamma-Glutamyl [U/L]** | 0.0% |
| **Sodium [mmol/L]** | 0.0% |
| **Chloride [mmol/L]** | 0.0% |
| **Total Protein [g/L]** | 0.0% |
| **Albumin [g/L]** | 0.0% |
| **Eosinophils [x10^9/L]** | 0.0% |
| **Lymphocytes [x10^9/L]** | 0.0% |
| **Basophils [x10^9/L]** | 0.0% |
| **Urine pH** | 0.0% |
| **Glucose Urine** | 0.0% |
| **Nitrites in Urine** | 0.0% |
| **Blood in Urine** | 0.0% |
| **HCG pregnancy test** | 0.0% |
