# Supplementary material for: Machine-learning based prediction of appendicitis for patients presenting with acute abdominal pain at the emergency department
Source: World J Emerg Surg. 2024 Dec 23;19:40. doi: 10.1186/s13017-024-00570-7 (PMC11664873; doi:10.1186/s13017-024-00570-7)
Supplement: Supplementary file 2 — Supplementary Material 2 [file 13017_2024_570_MOESM2_ESM.docx]

**Supplemental Materials**

This file provides additional information on the included patient population, the hyperparameter settings used for both the HIVE and HIVE-LAB model, as well as on the reader study which is supplemented with an example case (translated to English).

1. **Patient population details:**

**Age Distribution:**

o Pediatric Patients (≤10 years old): 25 patients, of whom 14 had appendicitis. Youngest was 4.

o Elderly Patients (≥65 years old): 91 patients; 27 had appendicitis. Oldest was 86.

**Pregnant Patients:**

o 5 patients were pregnant; 3 had appendicitis.

**Patients with Comorbidities:**

o Diabetes Mellitus: 11 patients; 4 had appendicitis.

o Crohn's Disease:

- 2 patients had Crohn's disease without appendicitis.
- 2 patients had a family history of Crohn's disease; 1 had appendicitis.

o Colitis: 1 patient had colitis without appendicitis.

1. **Hyperparameter settings XGBoost using Optuna hyperparameter optimization framework.**

For the HIVE model, the key critical hyperparameters were a learning rate (η) of 0.016, a maximum tree depth of 7, a minimum sum of weights in a child of 7, a subsample ratio of features for each tree of 0.94, and 740 boosting rounds. For the HIVE-LAB model, the settings included: a learning rate (η) of 0.038, a maximum tree depth of 8, a minimum sum of weights in a child of 10, a subsample ratio of features for each tree of 0.90, and 785 boosting rounds.

**Methodology Reader Study**

All 68 cases were uploaded to ResearchManager EDC for the reader study. Each of the 68 cases was independently reviewed by three emergency department (ED) physicians. The ED physicians had 1, 5, and 10 years of experience as practicing (not in residence) ED physicians.

The review process began with a Word document containing each patient's triage information, vital signs, anamnesis, and physical examination (see example case below). Initially, ED physicians assessed the likelihood of appendicitis on a scale from 0 to 100. Hereafter, laboratory test results were provided for each case, prompting physicians to reassess and, if necessary, revise their scores based on this additional information. Laboratory results were color-coded, blue for reduced values and red for elevated values, to aid interpretation and to mimic daily practice (see example below).

1. **Example Case 1 Word File**

Case 1

**Parameters**

| Age | 31 |
| --- | --- |
| Sex | F |
| Transport | Own Transport |
| Referrer | Primary Care Center |
| Number of ED visits | 1 |
| Oxygen saturation | 100 |
| Respiratory rate | nan |
| Blood pressure | 114/78 (90) |
| Heartbeat | 87.0 |
| EMV | nan |
| Temperature ºC | 37.4 |
| SIRS | 0.0 |
| qSOFA | 0.0 |
| Pain rating | 6.0 |

**Medical History & Physical Examination**

Medical History
History: Not contributing.

Medication: None
Allergies: furabid
Last meal: 17:00 frikandel (minced meat hot-dog)

Medical History:
Patient has been experiencing abdominal pain since last Thursday. Initially, the pain was periumbilical but migrated to the right lower quadrant (RLQ) after one day, with radiation to the back. The intensity of the pain varies. Migratory pain is present. Stool contains mucus admixture, and the patient has a history of variable defecation patterns. Today, there was an exacerbation of pain symptoms, prompting a contact with the primary care physician. The patient reports no nausea or vomiting. Abdominal distension is noted.

Tractus Urogenital: Last menstrual period occurred last Wednesday and was normal. There have been no recent changes in sexual contacts. No dysuria and hematuria.

Physical Examination:
General impression: Not acutely ill and is clear and alert.
Vital signs: Temp 37.4, H87, BP 114/78, Sat 100%
Abd: Slender abdomen. Hyperactive peristalsis. Variable tympanic without percussion pain. Diffuse tenderness with PM at McBurney. Right costovertebral angle tenderness.

**Example Case 1 Excel File**
